# Defect Tailored NiO Quantum Dots via Energy-Efficient Synthesis: Electronic Transport and Selective Cytotoxicity

**DOI:** 10.1021/acsomega.5c05954

**Published:** 2025-08-07

**Authors:** Vaishnavi K Mohan, Tanmayee Srinivas, Ansh Gupta, Vrushali Khedekar, Jordi Llorca, Teny Theresa John

**Affiliations:** a Department of Physics, 166231 Birla Institute of Technology and Science, Pilani, K K Birla Goa Campus, Zuarinagar, Goa 403726, India; b Department of Electrical and Electronics Engineering, Birla Institute of Technology and Science, Pilani, K K Birla Goa Campus, Zuarinagar, Sancoale, Goa 403726, India; c Department of Chemical Engineering and Barcelona Research center in Multiscale Science and Engineering, 16767 Universitat Politècnica de Catalunya, Barcelona 08019, Spain

## Abstract

Developing a cost-effective synthesis route for NiO at room temperature with a low calcination temperature (∼200 °C) remains a significant challenge. This study presents a novel, eco-friendly approach for synthesizing zero-dimensional NiO quantum dots (QDs) via a simple coprecipitation method using minimal reagents and energy-efficient processing. The resulting NiO QDs are obtained in powder form, enabling easy handling, storage, and integration into various applications. X-ray photoelectron spectroscopy, photoluminescence, and Raman spectra confirm the presence of interstitial oxygen (O_i_) and nickel vacancies (V_Ni_), indicative of intrinsic defects. Temperature-dependent conductivity analysis reveals two distinct regions separated by half the Debye temperature (θ_D_), suggesting the formation of small-polaron-like bound Zhang–Rice states. Furthermore, cytotoxicity studies conducted on A549 and HeLa cancer cell lines and L132 normal cells demonstrate selective toxicity toward cancer cells. These findings highlight the potential of defect-engineered NiO QDs for multifunctional applications, including optoelectronics and biomedicine.

## Graphical abstract

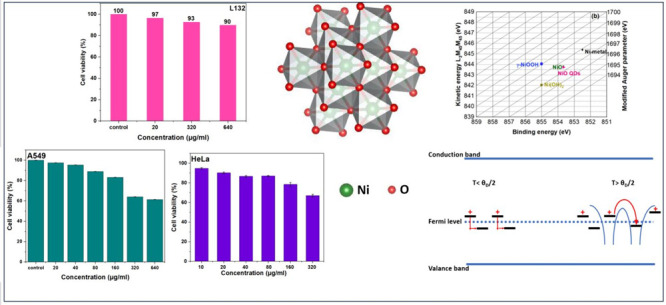

## 1 Introduction

In recent years, the synthesis and characterization of quantum dots (QDs) have garnered significant attention due to their unique optical, electronic, and catalytic properties, which hold promise for a wide range of applications, including solar cells, sensors, photocatalysis, and biomedical applications.^1–7^ Reducing particle size to the nanometer scale yields unique properties like quantum size effects, increased surface area, and low sintering temperatures. NiO stands out as a promising and environmentally friendly p-type binary semiconductor with a wide band gap energy, typically ranging from 3.6 to 4 eV in its bulk form. The versatility of NiO has led to extensive applications across various fields, including sensing devices,^8,9^ agriculture,^10^ spintronic devices,^11^ solar cells,^12,13^ supercapacitors,^14^ quantum tunneling,^15^ exchange-coupled dynamics,^16^ and optical coatings.^17^ Moreover, NiO nanomaterials exhibit exceptional thermal stability, rendering them suitable for applications in microelectronics and as electrochromic materials for displays.^18^ For instance, Zhang and colleagues investigated QD light-emitting diodes fabricated using a solution process, employing a hole injection layer composed of NiO nanocrystals.^15^ Notably, the electrical conductivity of NiO is influenced by intrinsic doping through the formation of microstructural defects attributed to V_Ni_ and interstitial oxygen (O_i_)^19^ or by extrinsic doping (e.g., group 1 elements).^20,21^ Early studies attributed DC conductivity in NiO to the small polaron hopping model, in which charge carriers (holes) are localized due to strong electron–phonon interactions and migrate via thermally activated hopping between adjacent sites. This mechanism has been widely used to describe charge transport in NiO. Karsthof et al. proposed an alternative explanation based on the polaronic interacceptor hopping model.^22^ In this framework, holes are not freely hopping between Ni^2+^ and Ni^3+^ sites but are strongly bound to V_Ni_, forming localized polaronic states. Charge transport occurs through thermally assisted hopping between these acceptor sites.^22^

Furthermore, the high surface area-to-volume ratio of NiO enables efficient therapeutic agent loading, while its nanoscale dimensions enhance penetration into tumor tissues. Additionally, the tunable emission wavelengths and low cytotoxicity of the NiO nanoparticles are suitable for long-term cellular imaging. The sensitivity to changes in the local environment, coupled with the fluorescence properties of NiO nanoparticles, enables the development of sensitive and selective biosensors for applications such as detecting biomarkers, monitoring drug concentrations, and diagnosing diseases. The application of NiO nanoparticles in photodynamic therapy against malignant cervical cancer cells has been reported.^23^ Selective laser activation of nanoparticles triggers necrosis in cancerous tissue by initiating a cellular damage mechanism. The production of reactive oxygen species (ROS) in NiO contributes to oxidative stress, which is responsible for the destruction of the DNA and mitochondria of the cancerous cells by inducing a false inflammatory response, ultimately leading to cell death via necrosis.^23^ Antibacterial and antifungal activities of NiO nanoparticles, of size ∼30 nm, were studied, leading to the disruption of the cell wall and inhibition of cell wall growth in bacteria and fungi.^24^ Adhikary and colleagues synthesized NiO from three distinct precursors and effectively employed it in drug delivery applications.^25^ They conjugated NiO with erythromycin and successfully delivered it against both Gram-positive and Gram-negative bacteria, presenting a promising strategy to overcome bacterial drug resistance in antibacterial therapy.^25^

Various NiO nanostructures were synthesized using hydrothermal,^14^ chemical precipitation,^26^ solvothermal processes,^27^ thermal decomposition,^28^ atomic layer deposition, and sol–gel^29^ techniques. For instance, the formation of nanodot wire and ring-like structures through the self-assembly of NiO QDs of size between 5 and 10 nm at 550 °C, in the presence of liquid crystal media, has been investigated for their optoelectronic characteristics.^30^ Similarly, atomic layer deposition of NiO at 400 °C has been reported as a route for getting a uniform layer of NiO. Venkatalakshmi et al. reported a green synthesis method for producing NiO nanoparticles with an approximate size of 17 nm, which were subsequently calcined at 400 °C.^31^ Nevertheless, many of these procedures demand prolonged protocols, elevated operating temperatures, or expensive equipment. Hence, it remains crucial to develop a simple procedure to synthesize NiO QDs using a low-temperature approach to exploit the remarkable properties of NiO across various application domains.

Here, we present a novel, simple, and cost-effective method for synthesizing NiO QDs in powder form with an average size of 3.5 nm, achieved through precipitation at room temperature, followed by low-temperature (∼200 °C) calcination for a controlled duration (1 h). We also provide a comprehensive characterization of the synthesized QDs, including their structural, morphological, and optical properties. PL emission analysis revealed defect-induced energy states, enabling estimation of the probable energy band structure of NiO QDs and identification of potential defect sites within the material. We analyzed the defect-induced DC conductivity of NiO using the polaronic interacceptor hopping mechanism, as the widely used free small polaron hopping model, originally proposed for high-purity samples, is unsuitable for intrinsically doped NiO. Additionally, the biocompatibility of the synthesized QDs was tested by the cytotoxicity assay using cancerous A549 (human lung cancer cell), HeLa (human cervical cancer cell), and normal L132 cell (lung embryonic cell) lines.

## 2 Materials and Methods

### 2.1 Experimental Details

NiO QDs were synthesized using analytical grade nickel acetate (Ni­(CH_3_COO)_2_, 98%) and sodium hydroxide (NaOH, 0.1 N standard solution purchased from Loba chemicals) from Sigma-Aldrich without further purification. An equimolar solution of NaOH was added to a 0.1 M solution of nickel acetate at room temperature with constant stirring, and the mixture was allowed to precipitate over 24 h. Subsequently, the mixture underwent washing and filtration, yielding a green-colored precipitate that was dried for 12 h at 60 °C in a hot air oven. The dried precipitate was then crushed and calcinated at 200 °C for 1 h (the minimum temperature required to form NiO, without any other impurity phases) to get NiO QDs in black color (low calcination temperature and time compared to the existing literature, Table 1). Figure 1 presents a schematic diagram illustrating the synthesis process of NiO QDs.

**Figure 1:**
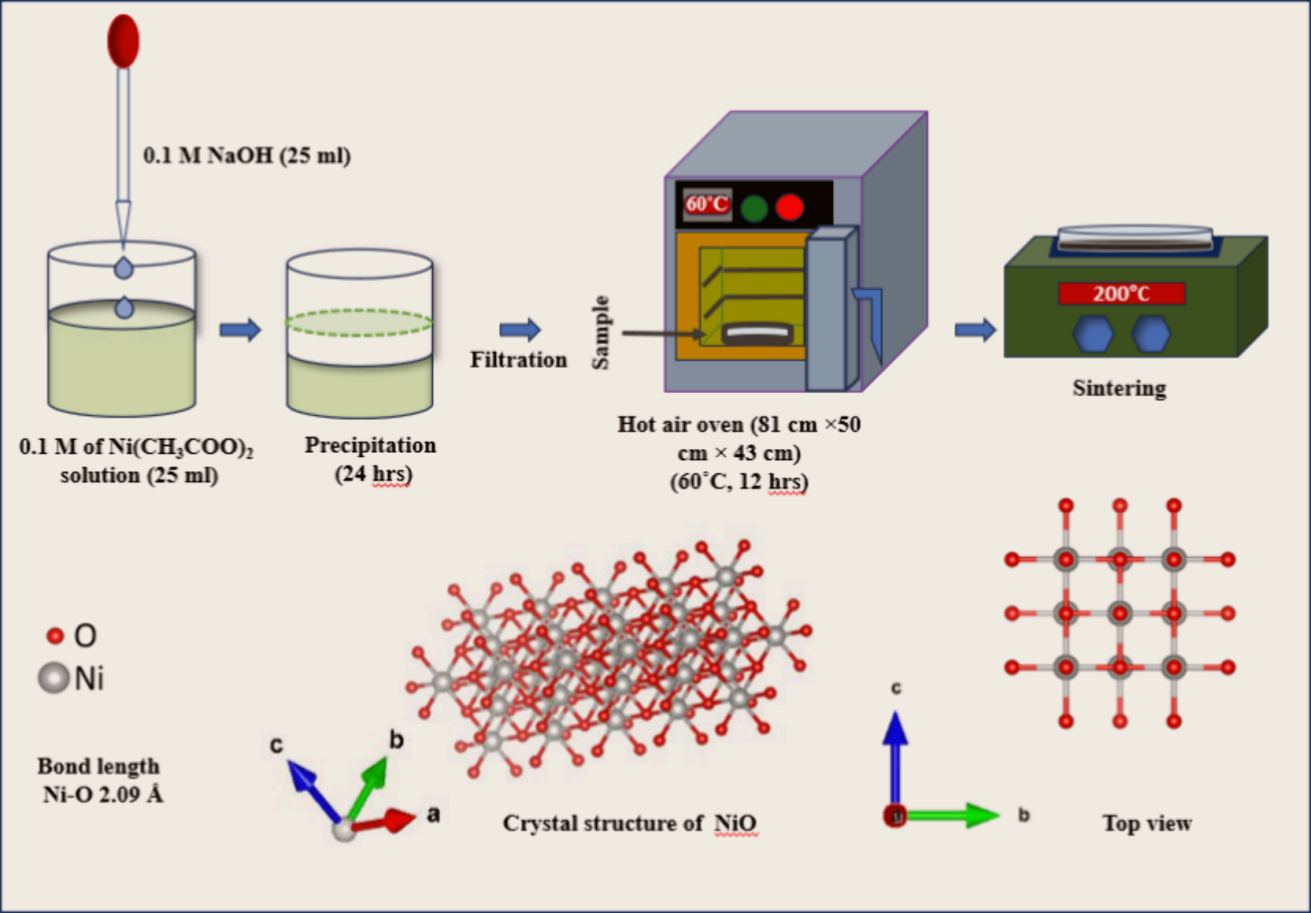
Schematic showing the synthesis of NiO QDs.

**Table 1:** Comparison of Our Synthesis Technique with the Existing Literature

**synthesis method**	**particle size (nm)**	**calcination temperature (°C)**	**calcination time (hours)**	**ref.**
solution processed	18	600	4	^32^
sol–gel	13–19	450	4	^33^
solution processed	27 (crystallite size)	300	2	^34^
solution processed	21	300	2	^35^
combustion method	22	500	4	^36^
pulsed laser deposition	∼30 (thin film)	600 (substrate temperature)		^22^
magnetron sputtering	∼5 (thin film)			^22^
hydrothermal	20–40	410	10	^37^
coprecipitation	3.5	200	1	this work

### 2.2 Characterization

X-ray Diffraction (XRD) of the NiO QDs is carried out using a Mini Flex II Rigaku X-ray diffractometer with a Cu K_α_ source (λ = 1.54 Å). The morphology and the particle size of the sample were determined by using transmission electron microscopy (TEM) (Jeol/JEM 2100). The elemental composition and mapping have been carried out using the (elemental dispersive spectroscopy) EDAX (Quanta FEI-450 FEG). The optical absorption of the sample was studied using UV–visible spectroscopy using a spectrophotometer (V-570, Jasco, Japan). Raman and PL spectra at room temperature were recorded using a LAB RAM HR Horiba France spectrometer with 532 and 325 nm laser sources. The Fourier Transform Infrared (FTIR) vibrational spectrum of the sample was obtained by using an IR Affinity-1 spectrophotometer (Shimadzu, Japan). XPS measurements were carried out using a SPECS system equipped with a PHOIBOS 150 EP hemispherical energy analyzer, an MCD-9 detector, and an XR-50 X-ray source operating at 150 W. Current–voltage characterization was carried out using a source measurement unit (SMU-2450). All the cell lines were cultured in a T25 flask in Dulbecco’s modified Eagle's media supplemented with 10% fetal bovine serum, 50 μg/mL Streptomycin, and 50 μg/mL penicillin (complete media) at 37 °C in a humidified atmosphere with 5% CO_2_ concentration. The cells were passaged at 80% confluency using 0.25% trypsin-disodium ethylenediaminetetraacetic acid (EDTA). For the MTT 3-(4,5-dimethylthiazol-2-yl)-2,5-diphenyltetrazolium bromide (MTT) assay, 65,000 cells were seeded on a 96-well plate and allowed to grow for 2 h. The NiO QDs were diluted in complete media and sonicated for 15 min before use. After 24 h postseeding, cells were treated with nanoparticle suspension with varying concentrations from 10 to 320 μg/mL or 640 μg/mL. and incubated for another 24 h. Subsequently, the wells were washed with phosphate-buffered saline (PBS), and 10 μL of MTT reagent (5 mg/mL) was added per well. Following incubation for 4 h at 37 °C in the dark, the formazan crystals were dissolved in 100 μL of dimethyl sulfoxide (DMSO). The percentage cell viability was determined by measuring the absorbance of the plates at 570 nm. The cell viability (%) was calculated as

$$\%\mathrm{cell}\;\mathrm{viability}=\left(\frac{A_{\mathrm{test}}}{A_{\mathrm{control}}}\right)\times 100$$ 1

where *A*_test_ is the absorbance of the cells exposed to nanoparticles and *A*_control_ is the absorbance of cells unexposed to nanoparticles. A two-tailed Student’s *t* test was used to analyze significant differences between the control and test groups.

## 3 Results and Discussions

### 3.1 Structural and Morphological Characteristics

Figure 2 shows the XRD pattern of NiO sample calcined at 200 °C. The pattern verifies that the sample has the face-centered cubic (FCC) structure with space group *Fm*3*m*, as indicated by the International Centre for Diffraction Data (ICDD) card number 04–0835 with preferred orientation along the (200) plane. From the pattern, NiO QDs are single-phase with no other impurity peaks. The broad XRD peaks suggest a small particle size of the sample. The average crystallite size and the strain of the NiO QDs are determined from the Williamson-Hall (W–H) plot, Figure S1 (Supporting Information). The average crystallite size (calculated from the Y-intercept of the W–H plot) is found to be ∼4 nm, which agrees with the value obtained from TEM. The negative slope (−0.001) yielded from the plot indicates compressive strain, suggesting lattice shrinkage due to the reduction in the particle size.^38^ Table 2 illustrates all the peaks, their corresponding Miller indices, and the interplanar separation (*d*).

**Figure 2:**
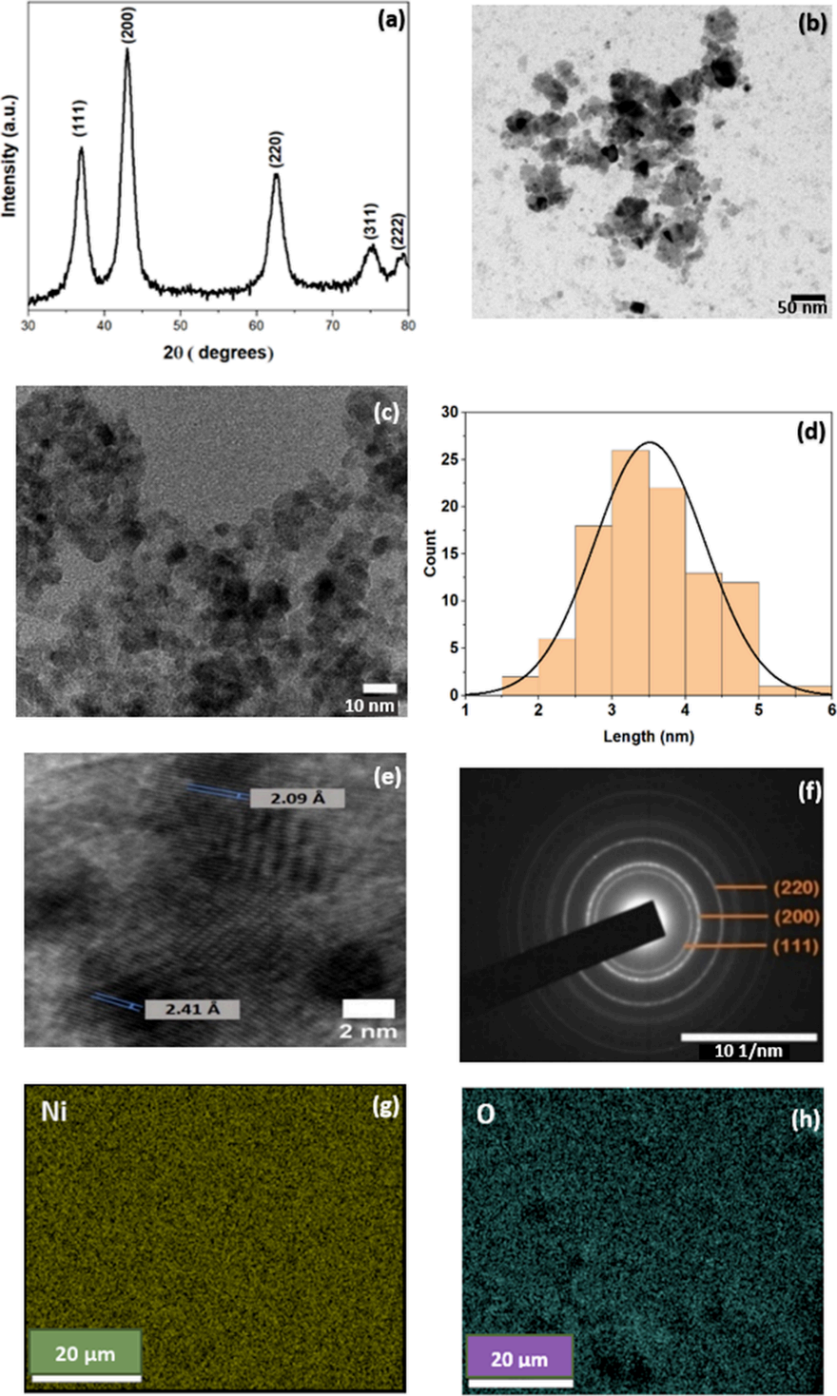
(a) XRD spectrum of NiO QDs corresponding to the ICDD card number 04–0835. TEM image of NiO QDs at a higher magnification showing the spherical-shaped particles. (b) 50 nm scale. (c) 10 nm scale. (d) Size distribution of NiO QDs with an average size of 3.5 nm. (e) High-resolution TEM image, at 2 nm scale with two preferential planes marked. (f) SAED pattern of NiO QDs with preferential growth along the (200) plane. Elemental mapping images of (g) Ni and (h) O.

**Table 2:** Comparison of the Experimental and Standard Values of Interplanar Separation Corresponding to Different Planes

**2θ** (**degrees**)	***hkl***	**experimental, *d* (** *Å* **)**	**ICDD, *d* (** *Å* **)**
37.27	111	2.41	2.41
43.29	200	2.09	2.08
62.91	220	1.48	1.47
75.44	311	1.25	1.25
79.39	222	1.20	1.20

The high-magnification TEM image of the NiO QD is depicted in Figure 2b,c. All of the QDs appear spherical, with an average size of 3.5 nm. The particle size distribution is given in Figure 2d. The particles are well dispersed with a small, narrow distribution of 1.5 to 6 nm. The high-resolution TEM image revealed interplanar spacings of 2.41 and 2.09 Å, corresponding to the (111) and (200) crystal planes of cubic NiO, as shown in Figure 2e.

The SAED image and the corresponding planes of NiO QDs are presented in Figure 2f. The multiple diffraction rings observed from the SAED indicate the polycrystalline nature of the material. Furthermore, the brightest ring in the SAED image confirms that preferential growth occurs along the (200) crystal plane, consistent with the XRD results. The atomic percentages of Ni and O are 48.7 and 51.3 from EDAX analysis (SI, Figure S2). No other elements were detected, suggesting a homogeneous distribution of Ni and O throughout the sample without any impurity. Corresponding elemental mapping images are given in Figure 2g,h. These characterization techniques collectively demonstrated the uniform morphology and exceptional phase purity of NiO QDs.

### 3.2 X-ray Photoelectron Spectroscopy

XPS is a powerful tool to understand the chemical composition and electronic states of the samples. Figure 3a,b depicts the Ni 2p and the O 1s spectra of NiO QDs. The XPS wide scan spectrum is given in the SI, Figure S3, which shows multiple peaks corresponding to different binding energies (BE) attributed to both Ni and O in the sample. The BE of the elements was calibrated with the C 1s signal (BE, 284.8 eV) as reference. The high-resolution XPS spectrum of Ni 2p is shown in Figure 3a. The double peak feature indicates the splitting of the spin–orbital core level of Ni 2p into Ni 2p_1/2_ and Ni 2p_3/2_. To evaluate this feature precisely, the Ni 2p XPS spectrum is deconvoluted into six Gaussian peaks and are named as A, B, C, D, E, and F. The BE values were assigned by comparing the fitted values with the National Institute of Standards and Technology (NIST) database and reported literature. Figure 3a displays three prominent peaks at 853.4 (A), 855.3 (B), and 871.7 eV (D), with corresponding satellites at 860.6 (C), 874 (E), and 878.9 eV (F). Peaks A and B are assigned to Ni 2p_3/2,_ while peak D corresponds to Ni 2p_1/2._ Peak C is the satellite of Ni 2p_3/2_, and peaks E and F are satellites of Ni 2p_1/2._ Based on binding energy values, peaks A, B, C, and D correspond to Ni^2+^ species,^19,39,40^ whereas the peaks at E and F represent Ni 2p_1/2_ with the Ni^3+^ valence state.^39–41^ It should be noted that distinguishing between Ni^2+^ and Ni^3+^ is challenging due to multiplet splitting and overlapping features. However, in the Ni 2p_1/2_ region, in addition to the expected Ni^2+^ peak at 871.7 eV, we observe a higher BE shoulder, which is suggestive of a minor Ni^3+^ contribution potentially arising from surface oxidation or nonstoichiometry.^42^

**Figure 3:**
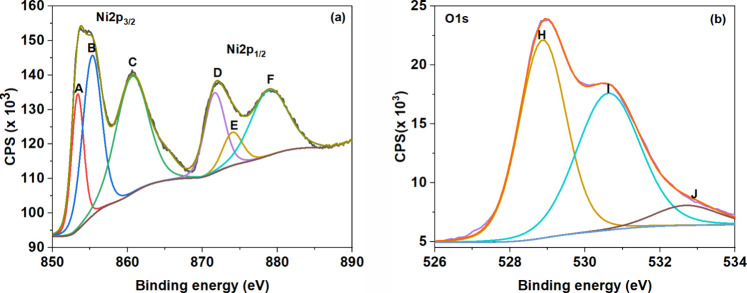
XPS spectra of NiO (a) Ni 2p and (b) O 1s.

NiO, a p-type semiconductor, inherently contains a significant concentration of V_Ni_. The formation of Ni^3+^ions arises from the oxidation of Ni^2+^ at these vacancy sites. Furthermore, the characteristic black color of NiO is attributed to the presence of V_Ni._ ^43^ To maintain charge neutrality in the vicinity of these vacancies, certain neighboring Ni^2+^ions undergo oxidation to Ni^3+^, facilitated by their interaction with the cationic hole (h^+^) generated by the initial oxidation of Ni^2+^. This process serves as a local charge-balancing mechanism without disrupting the crystalline structure of NiO.^19,39^ The crystal field distortion caused by the defects can split the energy levels, causing an increase in the band gap energy.^44^ Here, the spin–orbit splitting between Ni 2p_1/2_ and Ni 2p_3/2_ is 18.27 eV. This slight increase compared to the theoretical value (∼17.36 eV) indicates the coexistence of two oxidation states of Ni within the NiO.^45^

Similarly, the O 1s spectrum (Figure 3b) of NiO QD is deconvoluted to three Gaussian peaks centered around 528.9, 530.7, and 532.7 eV and labeled as H, I, and J, respectively. The peak H (528.9 eV) corresponds to O^2–^ ions associated with the Ni^2+^–O^2–^ bond in stoichiometric NiO and is referred to as lattice oxygen.^39–41,46^ Peak I (530.7 eV) is attributed to the presence of defect sites with lower oxygen coordination, commonly linked to Ni deficiency, which results in the formation of Ni^3+^ ions.^19,39^ Peak J (532.7 eV) is due to the surface adsorbed hydroxyl group in the sample.^39–41,46,47^

Auger lines are useful as an additional tool to identify the species present and distinguish between nickel oxides and nickel hydroxides. The Auger spectrum (Figure 4a) of NiO shows two well-resolved peaks characteristic of NiO. The shape and the position (the peak intensity of the Auger signal reaches its maximum at kinetic energy ∼843 eV) correspond to NiO and no other NiO phases, such as Ni­(OH)_2_ and γ–NiOOH.^48^

**Figure 4:**
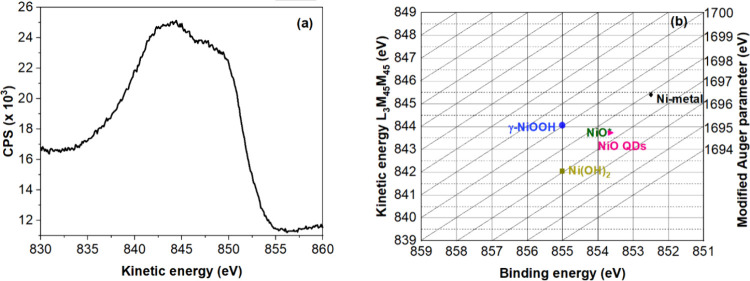
(a) Auger electron spectrum and (b) Wagner plot: the position of NiO QDs on the Ni 2p_3/2_-Ni LMM Wagner plot is consistent with the reference NiO. In the Figure, the synthesized quantum dots are referred to as NiO QDs and reference nickel oxide is represented as NiO.

The Wagner plot of Ni (Figure 4b), combining Ni 2p and L_3_M_45_M_45_ Auger information, is a graphical representation that plots the BE of Ni 2p_3/2_ against the kinetic energy of the L_3_M_45_M_45_ Auger peak.^48^ The right-hand *Y*-axis represents the modified Auger parameter, which is the sum of BE and kinetic energy in eV. The relative position of a sample on the Wagner plot can provide information about the chemical composition and oxidation state of the elements in the sample. In the plot, the synthesized NiO QD is denoted as NiO QDs and is compared against reference materials such as Ni metal, NiO, Ni­(OH)_2_, and γ-NiOOH.^48^ Elemental Ni displays the lowest BE for the Ni 2p_3/2_ peak on the Wagner plot. In contrast, NiO displays a higher binding energy compared with metallic Ni. Furthermore, NiO demonstrates a lower binding energy for the Ni 2p_3/2_ peak than other nickel compounds like Ni­(OH)_2_ or γ-NiOOH, indicating a lower oxidation state of nickel in NiO. The positioning of the NiO QDs in the Ni 2p_3/2_-Ni LMM Wagner plot, relative to Ni metal, reference NiO, Ni­(OH)_2_, and γ-NiOOH, confirms their identity as NiO.

### 3.3 Vibrational Spectroscopic Studies

The vibrational modes of NiO QDs were analyzed using Raman spectroscopy and the corresponding Raman spectrum is given in Figure 5a. The spectrum is dominated by a strong symmetric band spanning 300–700 cm^–1^. This can be deconvoluted into five peaks centered at 360, 455, 494, 541, and 572 cm^–1^. The deconvoluted peaks are shown in Figure 5b. The first peak at 360 cm^–1^ can be attributed to the TO phonon mode of vibration.^49^ The peak at 455 cm^–1^ represents the TO (Δ) mode.^49^ The Raman peak at 494 cm^–1^ is due to the LO (Δ) mode, a characteristic of crystalline NiO, which arises due to the nonstoichiometric Ni–O stretching.^49^ The peak at 541 cm^–1^ is assigned to the first order surface vibration modes, while that at 572 cm^–1^ represents the first order LO phonon mode, which indicates disorder induced by defects (V_Ni_) within the sample.^39,49,50^ This agrees with the observation of possible Ni^3+^ valence states in NiO (Figure 4a,b) formed due to the presence of V_Ni_. Furthermore, the small intensity peaks around 1040 and 1444 cm^–1^ correspond to 2LO phonon^50^ and 2M modes,^51^ respectively.

**Figure 5:**
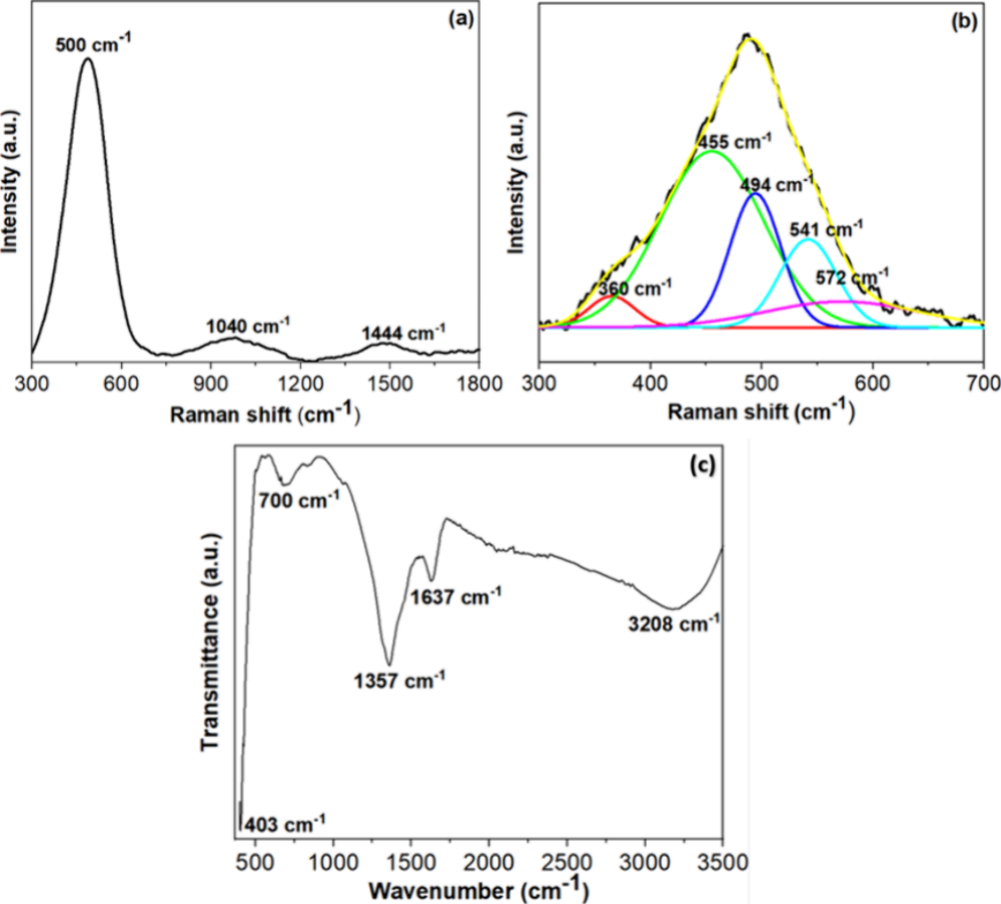
(a) Room-temperature Raman spectrum of NiO QD in the range of 300–1800 cm^–1^, (b) deconvoluted spectrum of the broad peak in the range of 300–700 cm^–1^. (c) FTIR spectrum of NiO QDs.

The low intensity of the 2LO mode indicates the small crystallite size of the particles, consistent with previous studies.^39^ The existence of longitudinal optical modes (LO and 2LO) suggests a strong interaction between Ni and O bonds.^39^ The 2M modes arise due to the antiferromagnetic superexchange interaction between the Ni and O ions in the linear atomic chain Ni^2+^–O^2–^–Ni^2+^.^51^

FTIR analysis of QDs serves as a valuable tool for detecting impurity molecules by identifying the chemical bonds in the sample. The FTIR spectrum is shown in Figure 5c. The spectrum exhibits a broad band around 403 and ∼700 cm^–1^. The band below 500 cm^–1^ represents the stretching mode of the Ni–O bond,^52,53^ while the band around 700 cm^–1^ arises due to the bending vibration of the metal–oxygen bond.^53^ Compared to bulk values (∼480 and 715 cm^–1^), the observed blueshift in the FTIR peak positions is attributed to the quantum size effect and the spherical shape of the QDs.^52^ The broad absorption band centered around 3208 cm^–1^ is assigned to the stretching mode of the hydroxyl group.^54^ The band at 1637 cm^–1^ corresponds to the H–O–H bending vibrations,^53^ and the one at 1357 cm^–1^ arises due to the C=O stretching vibration.^52^

### 3.5 Optoelectronic Studies

Figure 6a shows the room-temperature PL emission spectrum of the NiO QDs. A broad emission peak centered around 530 nm is observed in the visible region between 400 and 700 nm, indicating the presence of defects in the sample. The emission spectrum is deconvoluted into four Gaussian peaks that are categorized as emissions at violet (406 nm), green (530 nm), and red (647 and 734 nm). Figure 6b shows a schematic diagram of the defect levels and the corresponding emission of the NiO QDs. The violet emission corresponds to the transition of the electrons trapped at the Ni interstitials (I_Ni_) to the valence band.^43,55^ Compared with other emissions, the relatively small area under the peak associated with violet emission suggests a lower probability of I_Ni_ formation in the sample. The prominent green emission is attributed to electron transitions from V_Ni_ to the valence band.^43,56^ Both XPS and Raman measurements indicate the presence of V_Ni_ in the sample. NiO is commonly known as a metal-deficient binary oxide, where two Ni^2+^ ions react with excess oxygen to form ionized V_Ni_ to maintain the crystalline structure of NiO. Also, the reduced atomic percentage of Ni observed in the EDX analysis provides evidence for the presence of V_Ni_ within the sample. Conversely, the red emission is linked to the transition of electrons from the V_Ni_ to the O_i._ ^43,57^ The good electrical conductivity observed at room temperature (SI, Figure S4 and Figure 7) also indicates the presence of defect levels O_i_ and V_Ni._ ^19^ The emission spanning from violet to red in the visible spectrum, with a predominant green emission observed at room temperature, indicates the potential suitability of NiO for optoelectronic applications, including laser diodes, light-emitting diodes, and photodynamic therapy.

**Figure 6:**
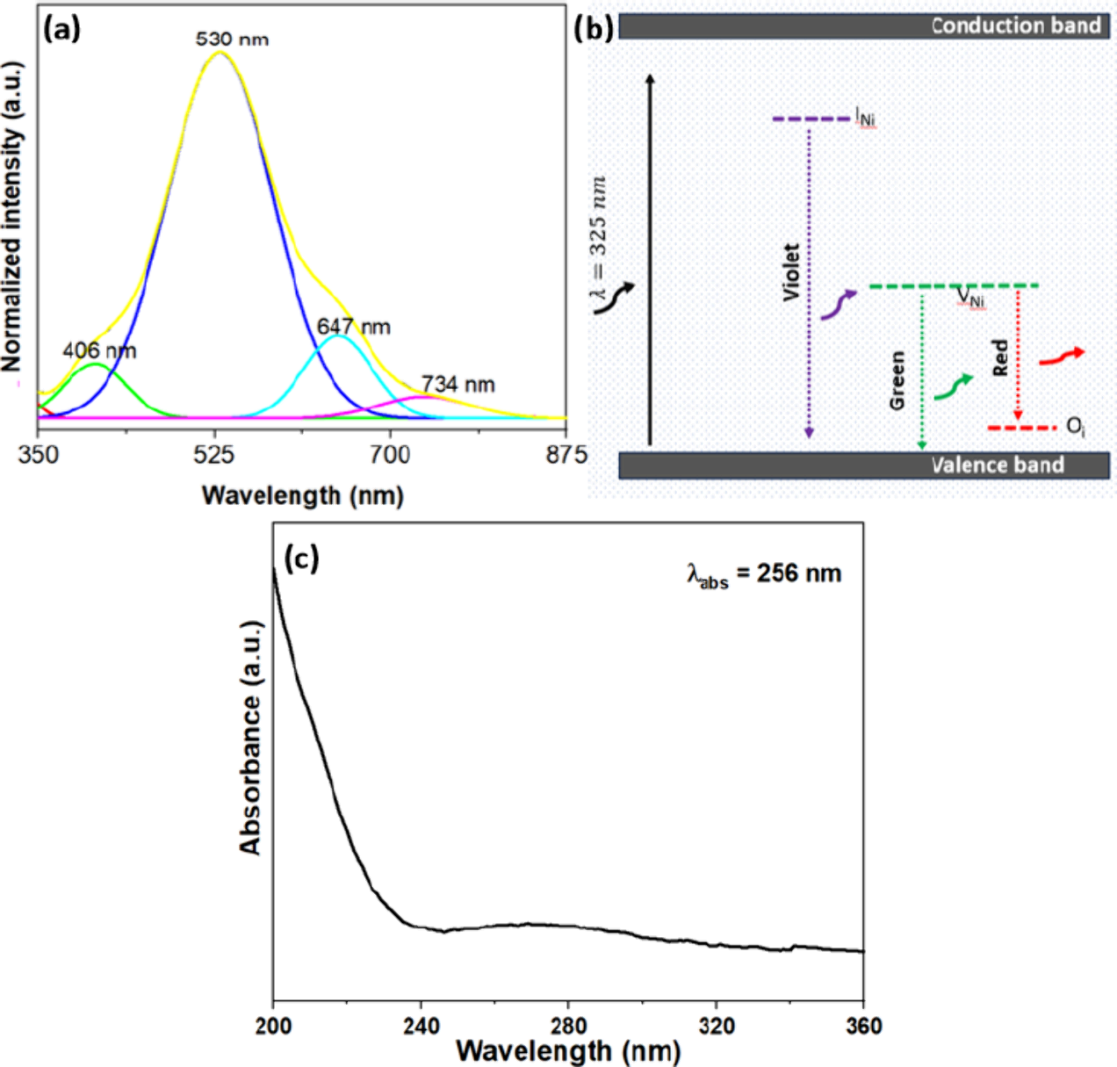
(a) Room-temperature PL emission spectrum of NiO QDs. (b) Schematic diagram showing the defect levels and corresponding emissions. (c) Absorbance spectrum of NiO QDs.

**Figure 7:**
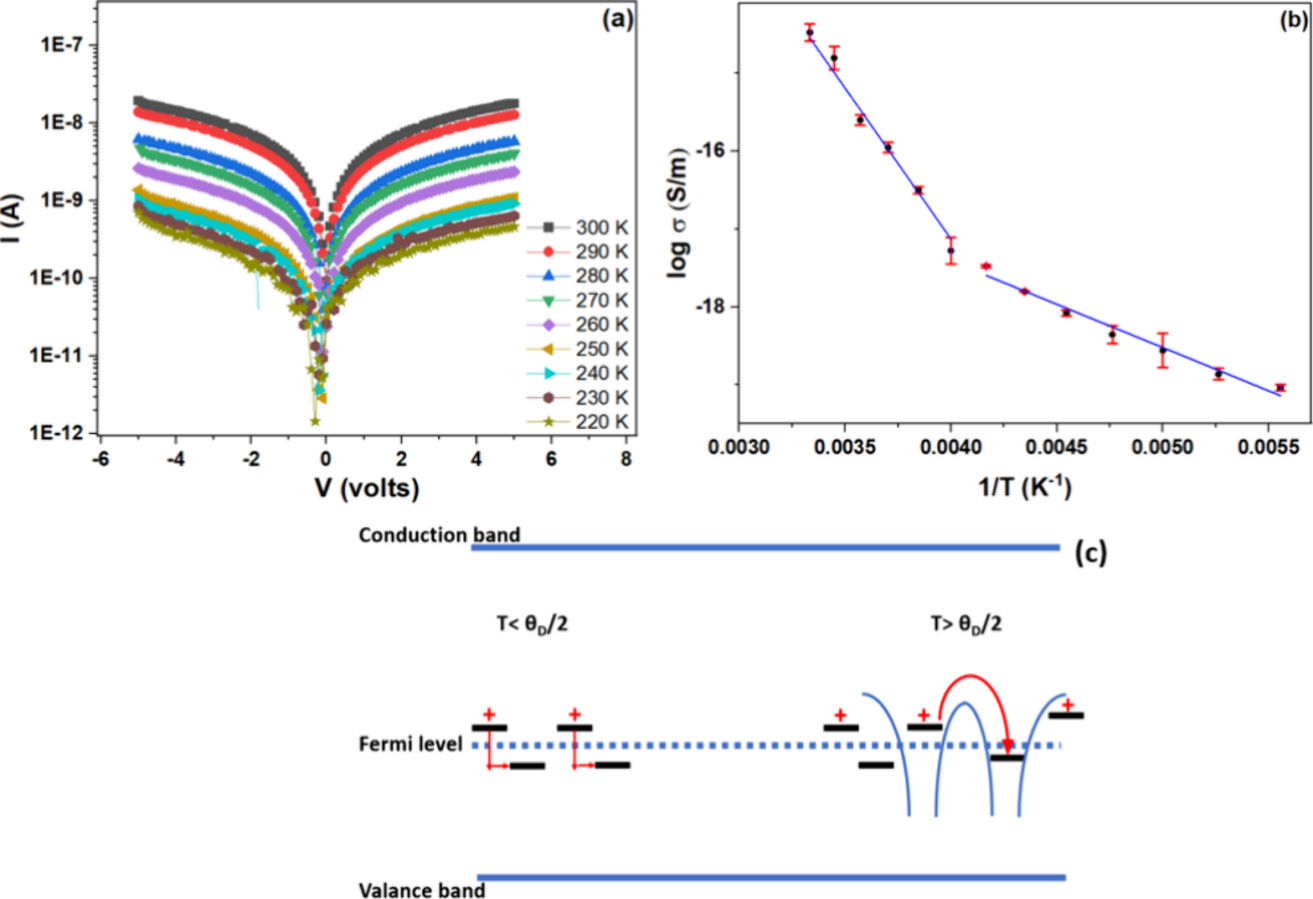
(a) Current–voltage characteristics on logarithmic scale for temperatures between 300 and 220 K. (b) Temperature dependence of the DC conductivity of NiO (obtained by numerical differentiation of the data from Figure 7) and fits according to the small polaron hopping model. (c) Schematic representation of the conduction mechanism in NiO both above and below $\frac{\theta_{D}}{2}$ . Spatial distances are not to scale.

Figure 6c demonstrates the UV–visible absorption spectrum of the NiO QD suspension obtained by ultrasonic dispersion in water. The NiO QDs exhibit a blue shift in the absorption onset. By extrapolating the linear region in the Tauc plot (SI, Figure S5), the band gap (direct) is found to be 4.84 eV.^58,59^ In semiconducting materials, the quantum confinement effect induces a notable blue shift in the absorption spectrum as the particle size decreases to nanoscale dimensions. The wider band gap compared to the bulk value is attributed to the quantum confinement effect.^58,60^

### 3.6 Transport Properties

We have carried out DC electrical conductivity of the sample with respect to the variation in the temperature (*T*) from 180 to 300 K. The transport properties of NiO QDs were studied by drop casting it between the two indium–tin oxide (ITO) electrodes. All measurements were conducted under a vacuum of 10^–5^ mbar. The variation of current with voltage (log I Vs V) is depicted in Figure 7a. The corresponding Arrhenius plot is given in Figure 7b. The reduction in the conductivity with respect to the decrease in the *T* suggests the semiconductor behavior of NiO. The Arrhenius plot shows a nonlinear behavior with two different slopes in the temperature range 300–250 and 240–180 K. One can also notice that these two temperature regions are separated at approximately 240 K, which is half of the Debye temperature (θ_*D*_) for NiO.^61,62^ In NiO, the temperature-dependent conductivity is explained based on free small polaron hopping. The polaron activation energy (*E*_*a*_) is quantitatively obtained using the conductivity equation,

$$\sigma \left(T\right)=\sigma_{0}e^{-E_{a}/kT}\mathrm{with}\sigma_{0}=\frac{Ne\vartheta a^{2}}{kT}$$ 2

where *N*, *e*, *a*, ϑ, and *k* are carrier density, charge of the carrier, lattice constant, optical phonon frequency, and Boltzmann constant, respectively.

The nonlinear behavior observed in the Arrhenius plot suggests that the small polaron hopping model is reproducible only for T ≥ ∼240 K ( $\frac{\theta_{D}}{2}$ ). The charge carriers hop over the energy barrier via multiphonon absorption at this temperature range. In our case, the polaronic activation energy obtained in the higher temperature region is 350 meV, which agrees with the already reported values.^22^ At *T* ≤ $\frac{\theta_{D}}{2}$ , the hopping over the barrier is suppressed, and the conductivity is dominated by polaronic interacceptor hopping by phonon-assisted tunneling through the barrier. Here, the holes are localized to the V_Ni_ (a polaron-like state) due to the formation of a ZR-bound state. The interaction between the hole spin and the majority 3d spin results in the formation of a ZR-bound state, exhibiting antiferromagnetic characteristics. Also, due to the above-mentioned strong localization and significant intersite spacing, band conduction remains suppressed. The antiferromagnetic behavior is supported by the mode at 1444 cm^–1^ from Raman spectroscopy (Figure 5a) and the M-H measurements (Figure S6). The conductivity exhibits a similar temperature dependence in this region as free small polaron hopping (eq 2), with a noticeable decrease in *E*_*a*_ (∼90 meV). Here, *E*_*a*_ represents activation energy comparable to the average energy separation between adjacent defect sites.^22^ This polaronic interacceptor hopping has previously been applied to Li-doped NiO^20,21^ and can also be extended to intrinsically V_Ni_-doped NiO. So, V_Ni_ acts as the acceptor level here, in the place of Li for Li-doped NiO. Figure 7c. represents the schematic representation of the conduction mechanism in NiO, both above and below $\frac{\theta_{D}}{2}$ .

### 3.7 Cytotoxicity Studies

The in vitro cytotoxicity of NiO QDs was comprehensively evaluated by simultaneously treating both cancerous (A549 and HeLa) and normal (L132) cell lines with various concentrations of NiO QDs for 24 h. The results, given in Figure 8, provide a comparative analysis of the cytotoxic effects across these cell lines using the MTT assay and their corresponding bright-field microscopy images before and after the treatment with NiO QDs.

**Figure 8:**
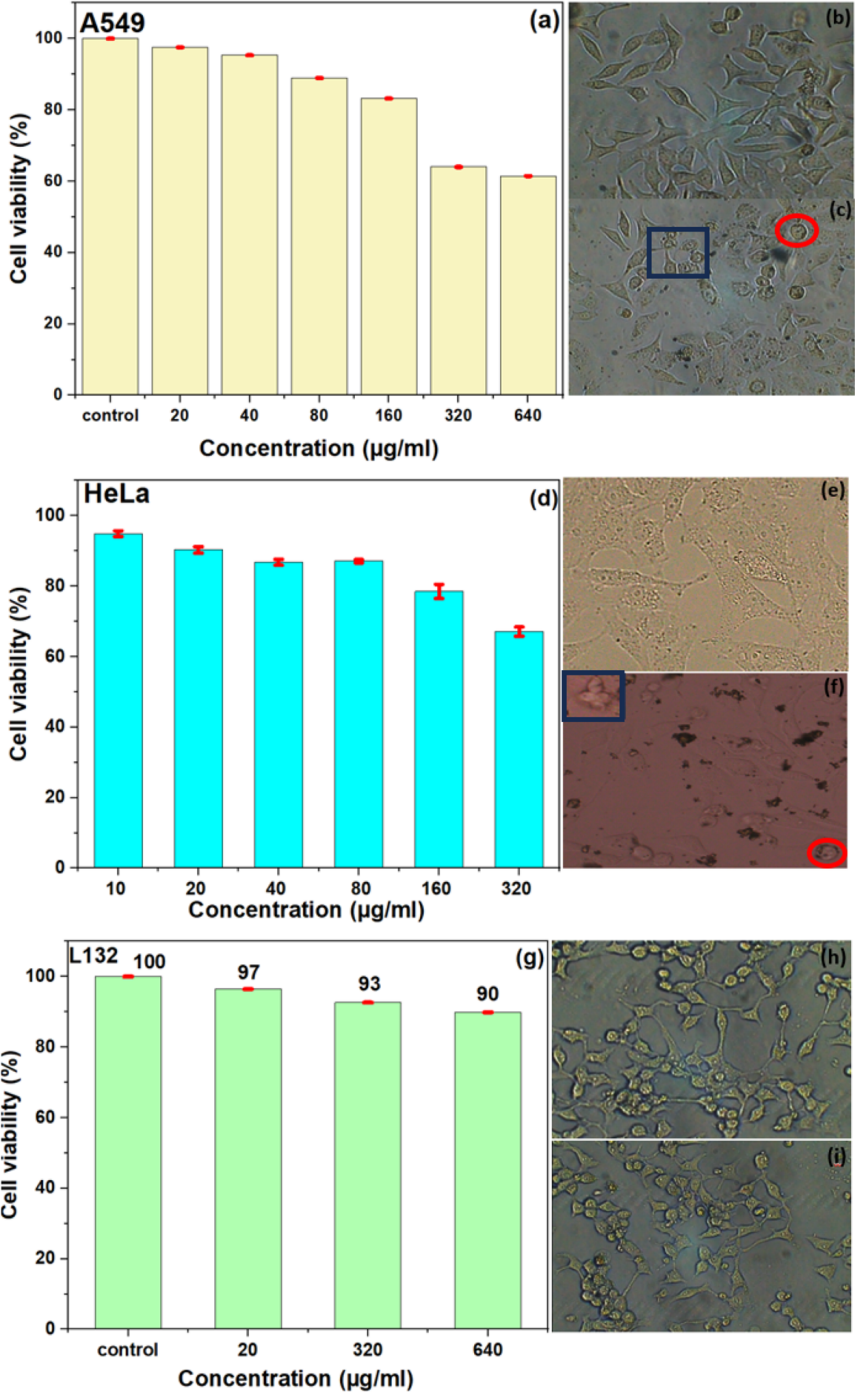
Cytotoxicity analysis of different cell lines by MTT assay after incubation with different concentrations of NiO QDs for 24 h (a) A549 cells. Optical microscopic images of (b) untreated A549 and (c) A59 cells incubated for 24 h with 640 μg/mL NiO QD obtained at 20× magnification. (d) HeLa cells. Optical microscopic images of (e) untreated HeLa cells. (f) HeLa cells incubated for 24 h with 320 μg/mL NiO QD obtained at 20× magnification. (g) L132 cell lines. Optical microscopic images of (h) untreated L132 cells and (c) L132 cells incubated for 24 h with 640 μg/mL NiO QD obtained at 20× magnification. The treated groups demonstrate statistically significant variances from the control group, as confirmed by Student’s *t* test (*P* < 0.05).

In A549 cancer cells (Figure 8a), viability remained relatively high at lower concentrations (20–80 μg/mL), ranging from approximately 97% to 88%. However, a moderate decrease to 83% viability was observed at 160 μg/mL, followed by a notable reduction to 64% at 320 μg/mL and further decline to 60% at 640 μg/mL. Similarly, HeLa cells (Figure 8d) exhibited high viability (95 to 88%) at lower concentrations (10–80 μg/mL), with a noticeable decrease to 78% at 160 μg/mL and a further reduction to 68% at 320 μg/mL. This cell viability remains notably higher than previously reported values in similar studies.^34,63–65^ These findings indicate that NiO QDs induce a dose-dependent cytotoxic effect in cancerous cells.

Cancer cells have a negative surface charge compared to normal cells.^66^ The coexistence of two different cationic states (Ni^2+^ and Ni^3+^) in NiO QD can induce a strong electrostatic force of attraction toward cancer cells, helping with their cellular uptake. Once internalized, NiO QDs induce oxidative stress through various mechanisms, disrupting cellular functions and reducing cell viability. The unique electronic properties of NiO facilitate charge transfer, generating ROS like hydroxyl radical (OH•), superoxide anions (O_2_^–^•), and hydrogen peroxide (H_2_O_2_).^67,68^ When holes (h^+^) combine with the hydroxyl groups (H_2_O, OH^–^), they become OH•, while the electron reacts with the O to produce O_2_^–^•. These intermediates further facilitate the formation of H_2_O_2_ and additional OH•,^57^ enhancing oxidative stress.

XPS analysis confirms the presence of Ni^2+^ and Ni^3+^ ions within the NiO matrix, with the formation of Ni^3+^ ions attributed to V_Ni_. Raman and conductivity measurements further support this nonstoichiometric composition. Meanwhile, FTIR and XPS-O 1s measurements confirm the presence of surface adsorbed hydroxyl groups. Cancer cells have a higher basal level of ROS as compared to normal cells. Incubation of NiO QD further increases the intracellular ROS level, resulting in higher levels of ROS that lead to cell death induced by DNA damage.^69^ After 24 h of treatment with NiO QDs, some cells began to show signs of distress, losing their original shape. Additionally, the presence of apoptotic bodies suggests that exposure to NiO QDs triggers apoptotic pathways in the cells. As marked in Figure 8c,f, certain cells began to shrink and detach from the surface, with red circles marking dead cells and blue rectangles highlighting detached cells.

Conversely, the normal L132 cell line exhibited minimal cytotoxicity (Figure 8g), with no significant reduction in cell viability observed, even at higher concentrations of NiO QDs (20–640 μg/mL). This stability in normal cell viability across all tested concentrations highlights the selective cytotoxic nature of NiO QDs toward cancerous cells. A comparative study on the cytotoxicity of various compounds, including metal oxides and other nanoparticles (Table 3), confirms that the sythesized NiO QDs exhibit minimal cytotoxicity toward normal cells, even at higher concentrations. Notably, after 24 h of treatment with 640 μg/mL, cell viability remains around 90%. In contrast, similar studies report significantly lower safe exposure limits.

**Table 3:** Comparative Study On the Cytotoxicity of Various Compounds, Including Metal Oxides and Other Nanoparticles on Normal Cells

**compound/particle**	**normal cell line**	**safe-level dose suggested (μg/mL)**	**cell viability (%)**	**exposure time (hours)**	**ref.**
ZnO (chemical synthesis)	Vero	0.05	>90	24	^68^
ZnO (green synthesis)	Vero	0.39	>90	24	^68^
ZnO	human fibro blasts	10	>80	24	^70^
Pt	peripheral blood mononucleocyte	200	>90	48	^71^
nonporous SiO_2_	mast	100	∼70	24	^72^
porous SiO_2_	mast	100	>90	24	^72^
TiO_2_	mast	100	>90	24	^72^
CeO_2_	lymphocytes	10	>90	24	^73^
NiO	epithelial	1 mg/mL	∼60	24	^74^
NiO	L132	640	∼90	24	this work

The selective cytotoxicity of NiO QDs enables their effective role in directly eliminating cancer cells while also enhancing their potential as drug delivery carriers. By combining their inherent anticancer activity with the ability to transport therapeutic agents, NiO QDs can improve treatment efficacy while reducing adverse effects on healthy tissues.

The nonstoichiometric composition achieved through controlling the synthesis parameters modulates the electronic structure, enhances the transport properties while simultaneously enabling selective interactions with cellular environment for targeted cytotoxicity.

## 4 Conclusions

In summary, we have successfully deposited NiO QDs employing a facile, low-cost technique at a low temperature through a simple solution-based process. The particle size is 3.5 nm from TEM, indicating confinement of the dimensions. The quantum confinement widens the band gap and is found to be 4.84 eV. XPS analysis of the Ni 2p spectrum confirms the coexistence of Ni^2+^ and Ni^3+^ ions within the sample. The presence of Ni^3+^ ions originates from the oxidation of Ni^2+^ at V_Ni_, a finding further supported by Raman measurements. A corresponding emission associated with V_Ni_ is also observed in the PL measurements. The O 1s spectrum confirms the Ni^2+^-O^2–^ bonding characteristic of NiO, as corroborated by Auger electron spectroscopy, Raman spectroscopy, and FTIR analysis. The temperature-dependent behavior of DC conductivity in NiO can be attributed to the polaronic interacceptor hopping mechanism. The structural and electronic disorder significantly influences the conduction process, where holes become localized at nickel vacancies, forming polaron-like states. These localized states facilitate charge transport by hopping between adjacent acceptors. This conduction mechanism is distinctly marked by two temperature regions, separated by approximately half the Debye temperature within the range of 250–200 K. The high cell viability (∼90%) observed in normal cells, L132, even at elevated concentrations (640 μg/mL for 24 h), along with the dose-dependent reduction in cancer cell viability (∼64–68% for both A549 and HeLa), demonstrates the selective cytotoxicity of NiO QDs, highlighting their potential for applications such as drug delivery, bioimaging and biosensing. The comparison of the effect of NiO QDs on cytotoxicity between the normal and cancer cell lines exhibits selective cytotoxicity toward cancer cells while sparing normal cells. This property enables NiO QDs to directly induce toxic effects on cancer cells, further enhancing their potential as drug delivery carriers.

## Supplementary Material



## Notes

W–H plot, EDAX, wide scan survey, room-temperature *I*–*V* curve, Tauc plot, and M-H plot of NiO QDs (PDF)
