# Supplementary material for: Defect Tailored NiO Quantum Dots via Energy-Efficient Synthesis: Electronic Transport and Selective Cytotoxicity
Source: ACS Omega. 2025 Aug 7;10(32):36697–707. doi: 10.1021/acsomega.5c05954 (PMC12368698; doi:10.1021/acsomega.5c05954)
Supplement: Supplementary file 1 [file ao5c05954_si_001.pdf]

SUPPLEMENTARY INFORMATION

Defect tailored NiO quantum dots via energy efficient synthesis: electronic transport and selective cytotoxicity

Vaishnavi K Mohan[a], Tanmayee Srinivas[a], Ansh Gupta[b], Vrushali Khedekar[a], Jordi Llorca[c] and Teny Theresa John[a*]

[a] Department of Physics, Birla Institute of Technology and Science, Pilani, K K Birla Goa Campus, Zuarinagar, Sancoale, Goa 403726, India

[b] Department of Electrical and Electronics Engineering, Birla Institute of Technology and Science, Pilani, K K Birla Goa Campus, Zuarinagar, Sancoale, Goa 403726, India

[c] Department of Chemical Engineering and Barcelona Research center in Multiscale Science and Engineering, Universitat Politècnica de Catalunya, Barcelona, Spain

E-mail: teny@goa.bits-pilani.ac.in

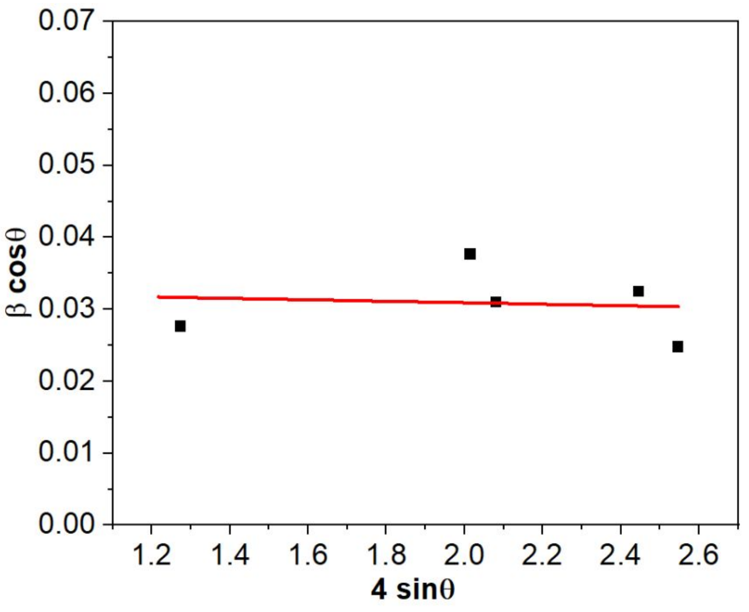

Fig. S1. W-H Plot of NiO QDs shows a slope of -0.001.

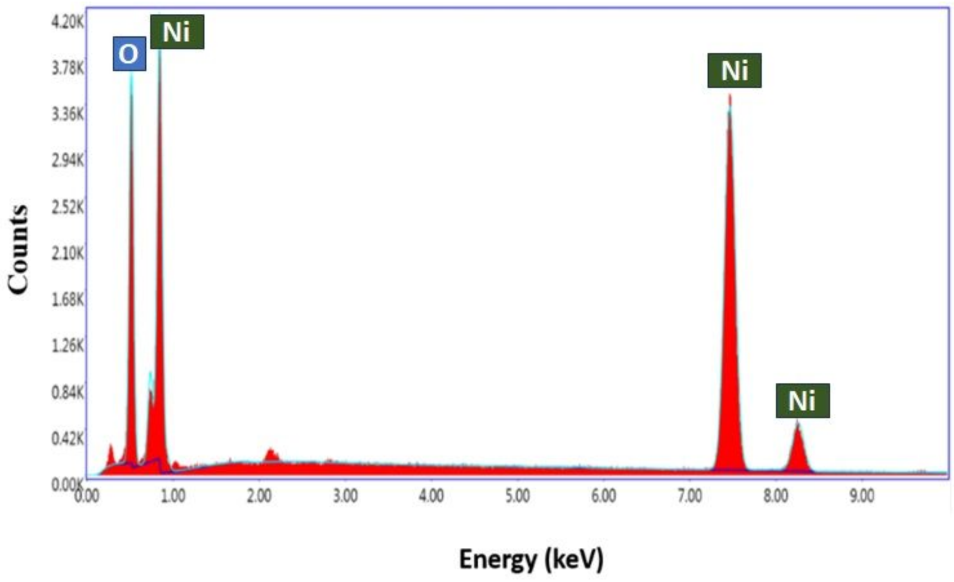

Fig. S2. Elemental composition of NiO QDs from EDAX.

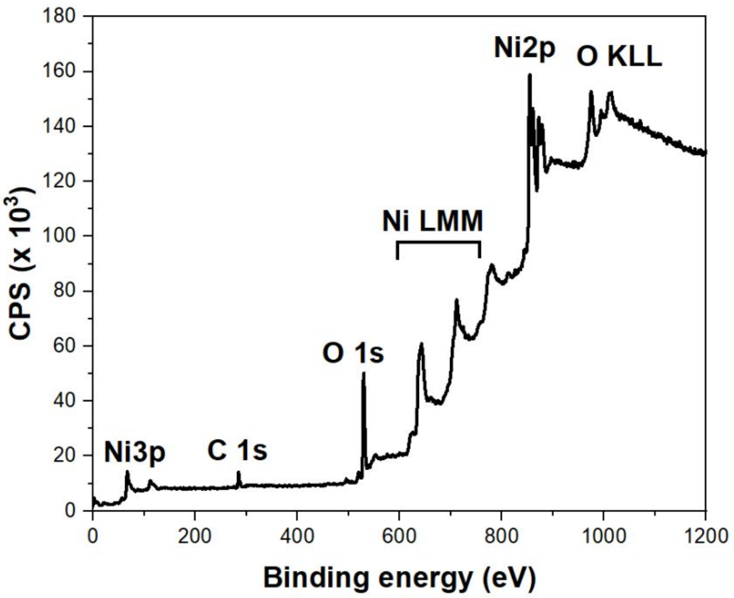

Fig. S3. The wide survey scan of NiO QDs.

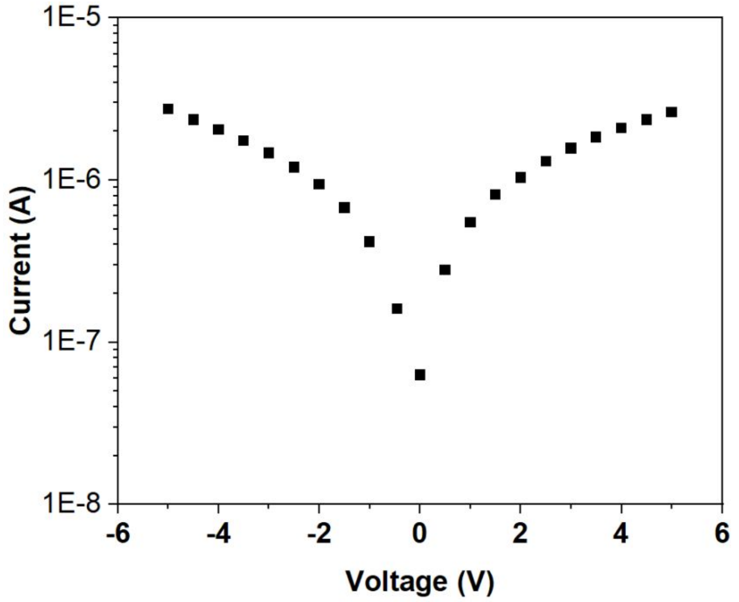

Fig. S4. The I-V curve of NiO QDs shows an enhanced conductivity at room temperature.

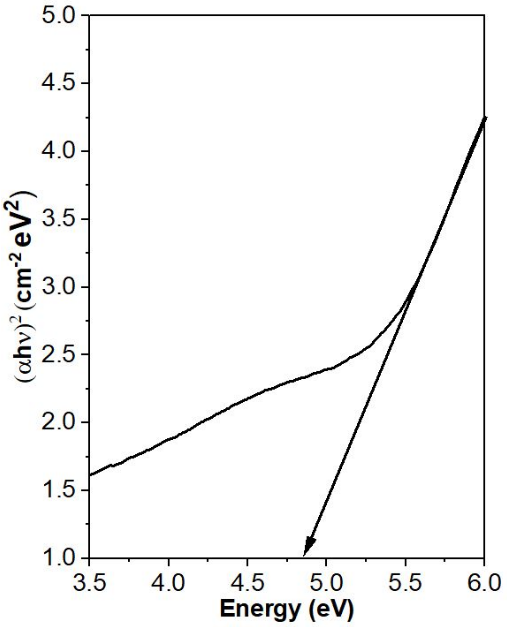

Fig. S5. Tauc plot of NiO QDs.

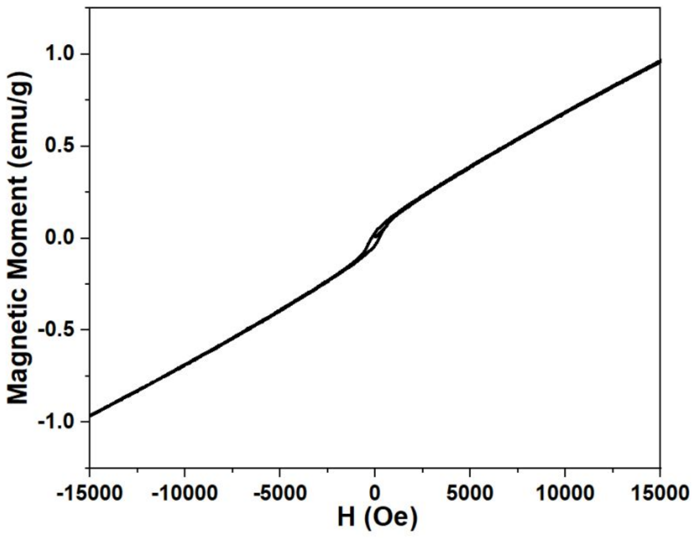

Fig. S6. M-H of NiO shows the antiferromagnetic behaviour at room temperature.
